# Safety and efficacy of tiotropium Respimat versus HandiHaler in patients naive to treatment with inhaled anticholinergics: a *post hoc* analysis of the TIOSPIR trial

**DOI:** 10.1038/npjpcrm.2015.67

**Published:** 2015-11-05

**Authors:** Robert Wise, Peter MA Calverley, Ronald Dahl, Daniel Dusser, Norbert Metzdorf, Achim Müller, Andy Fowler, Antonio Anzueto

**Affiliations:** 1 Department of Medicine, Johns Hopkins University School of Medicine, Baltimore, MD, USA; 2 Respiratory Medicine, Institute of Ageing and Chronic Disease, University of Liverpool, Liverpool, UK; 3 Allergy Centre, Odense University Hospital, Odense C, Denmark; 4 Service de Pneumologie, Hôpital Cochin, AP-HP, Université Paris Descartes, Sorbonne Paris Cité, Paris, France; 5 Medical Affairs, Boehringer Ingelheim Pharma GmbH & Co KG, Ingelheim, Germany; 6 Global Clinical Operations, Biometry and Data Management, Boehringer Ingelheim Pharma GmbH & Co KG, Biberach, Germany; 7 Medical Affairs, Boehringer Ingelheim Pharma Ltd, Bracknell, UK; 8 Department of Medicine, Pulmonary Critical Care Center, University of Texas Health Science Center, San Antonio, TX, USA

## Abstract

**Background:**

Patients with chronic obstructive pulmonary disease (COPD) who were naive to anticholinergics before the TIOtropium Safety and Performance In Respimat (TIOSPIR) trial may reflect patients seen in practice, in particular in primary care. In addition, investigating safety in these patients avoids the potential bias in patients who previously received anticholinergics and may be tolerant of their effects.

**Aims:**

The aim of this study was to evaluate whether patients naive to anticholinergic therapy who were treated with tiotropium Respimat 2.5 or 5 μg had different safety and efficacy outcomes than patients treated with tiotropium HandiHaler 18 μg.

**Methods:**

*A post hoc* analysis of patients who were not receiving anticholinergics before TIOSPIR (*N*=6,966/17,135) was conducted. Primary end points were risk of death from any cause and risk of COPD exacerbation. Secondary outcomes included severe exacerbation and major adverse cardiovascular events (MACE). Additional analysis of exacerbations was carried out in anticholinergic-naive patients with moderate (GOLD II) disease.

**Results:**

Anticholinergic-naive patients had less severe disease than the total TIOSPIR population. Discontinuations because of anticholinergic side effects were infrequent (0.9% overall). Similar to the primary study, patients in the tiotropium Respimat groups had no difference in the risk of death or risk of any or severe exacerbation than patients treated with tiotropium HandiHaler. Risk of MACE was similar across the Respimat and HandiHaler groups. Rates of exacerbations in the subgroup of patients with moderate disease were similar across the Respimat and HandiHaler groups.

**Conclusions:**

Tiotropium Respimat and HandiHaler have similar safety and efficacy profiles in patients who are naive to anticholinergic therapy.

## Introduction

Tiotropium (SPIRIVA, Boehringer Ingelheim Pharma & Co KG, Ingelheim am Rhein, Germany) is a once-daily, long-acting, anticholinergic bronchodilator available in two formulations: dry powder via HandiHaler (18 μg once daily) and as an aqueous solution via the Respimat Soft Mist Inhaler (5 μg (two puffs of 2.5 μg once daily)).^1,2^ Tiotropium HandiHaler 18 μg and tiotropium Respimat 5 μg have demonstrated similar improvements in lung function, symptoms and quality of life^3–6^ and have similar pharmacokinetic profiles in patients with chronic obstructive pulmonary disease (COPD).^7^

Queries around the safety of Respimat^8–10^ were addressed in the TIOtropium Safety and Performance In Respimat (TIOSPIR; NCT01126437) trial^11^—the largest long-term, randomised, double-blind trial in patients with COPD performed to date. The trial showed similar safety and exacerbation efficacy profiles for tiotropium Respimat 2.5 μg and 5 μg compared with HandiHaler 18 μg.

A common criticism of randomised clinical trials (RCTs) is that the selected patient populations may not be truly representative of patients seen in routine clinical practice—in particular, patients treated in primary care.^12–14^ A unique characteristic of TIOSPIR was the liberal inclusion criteria that were chosen to select a typical COPD patient population, including patients with a wide range of disease severities, as well as patients with stable cardiac disease.^11^

TIOSPIR also included a large population of patients with COPD who were naive to anticholinergic treatment at baseline. This represents an important patient group that may be highly representative of patients commonly seen in primary care. Patients within the primary TIOSPIR study who were previously treated with anticholinergics might be seen to constitute a selected population with a higher tolerance to anticholinergic effects and could have introduced a selection bias in the analysis of tiotropium’s safety. Therefore, it is conceivable that the anticholinergic-naive group might have been at an increased risk of side effects commonly associated with the class and may have responded differently to treatment with tiotropium Respimat or HandiHaler.

To investigate this possibility, this *post hoc* analysis of the TIOSPIR trial studied whether patients who had not previously received anticholinergic therapy and who were treated with Respimat 2.5 or 5 μg had different safety and/or efficacy outcomes compared with patients treated with HandiHaler 18 μg.

## Materials and methods

### Study design

TIOSPIR was a large (*N*=17,135) long-term (2–3 years), randomised, double-blind, double-dummy, parallel-group, actively controlled, event-driven trial in patients with COPD.^11,15^ Patients received once-daily Respimat 2.5 μg (two puffs of 1.25 μg) or 5 μg (two puffs of 2.5 μg) or once-daily HandiHaler 18 μg. Primary outcomes were time to death from any cause and time to first COPD exacerbation. Secondary outcomes included time to severe (hospitalised) exacerbations and time to major adverse cardiovascular events (MACE). The study design has been described previously.^11,15^

### Study population

Patients had a diagnosis of COPD with forced expiratory volume in 1 s (FEV_1_)⩽70% of forced vital capacity, FEV_1_⩽70% predicted and ⩾10 pack-years of smoking history, were aged ⩾40 years and were permitted to use their usual background treatment for COPD other than anticholinergics. All patients (including those with premature discontinuation) were followed up for vital status until the end of the study.

At enrolment, patients were asked to provide details of COPD and other medication received within the past 2 months. For this *post hoc* subgroup analysis, patients were included if they were not receiving short- or long-acting inhaled anticholinergics during the 2 months before the start of the trial.

### Statistical analysis

Hazard ratios (HR) and 95% confidence intervals (CI) were calculated using a Cox-proportional hazards regression model (without covariate adjustment). Rate ratios and 95% CI were used to compare incidence rates. Negative binomial regression models were used to compare event rates.

For analysis of death (including fatal MACE), events occurring during treatment and vital status follow-up (vital status analysis) were considered. For all other analyses (including MACE and COPD exacerbations), only events with onset in the on-treatment period were noted (on-treatment analysis). An on-treatment sensitivity analysis was conducted for time to death. Subgroup analyses by patient characteristics at baseline were conducted for time to death and exacerbation risk.

## Results

### Study population

Overall, 6,966 patients naive to anticholinergic treatment at baseline (Respimat 2.5 μg *n*=2,345; Respimat 5 μg *n*=2,312; HandiHaler 18 μg *n*=2,309) were randomised and treated for a mean follow-up duration of 834 days. A total of 541 (23.1%), 517 (22.4%) and 500 (21.7%) patients in the Respimat 2.5 μg, 5 μg and HandiHaler 18 μg groups, respectively, prematurely discontinued from the study. Discontinuation attributed to anticholinergic side effects occurred in 0.7 and 1.0% of patients in the Respimat 2.5 and 5 μg groups, respectively, and 0.9% of patients in the HandiHaler group.

Patient baseline demographics and characteristics were similar in the three groups (Table 1). Most patients were Global Initiative for Chronic Obstructive Lung Disease (GOLD) stages II (48.8%) and III (38.4%). The majority of patients (77.3%) were receiving pulmonary medication at baseline; approximately half of the patients were taking an inhaled corticosteroid or long-acting β_2_-agonist (50.2 and 51.6%, respectively) (Table 1). Of the patients who reported symptoms of breathlessness (*n*=6,688; 96.1%), the majority were classified as modified Medical Research Council (mMRC) scale 1 (*n*=2,694, 40.3%) or scale 2 (*n*=2,486, 37.2%) (Table 1). Approximately 8–9% of patients had a history of cardiac arrhythmia (Table 1). A total of 3,349 (48.1%) patients had ⩾1 COPD exacerbation in the year before the study (Table 1).

### Safety

**Figure 1:**
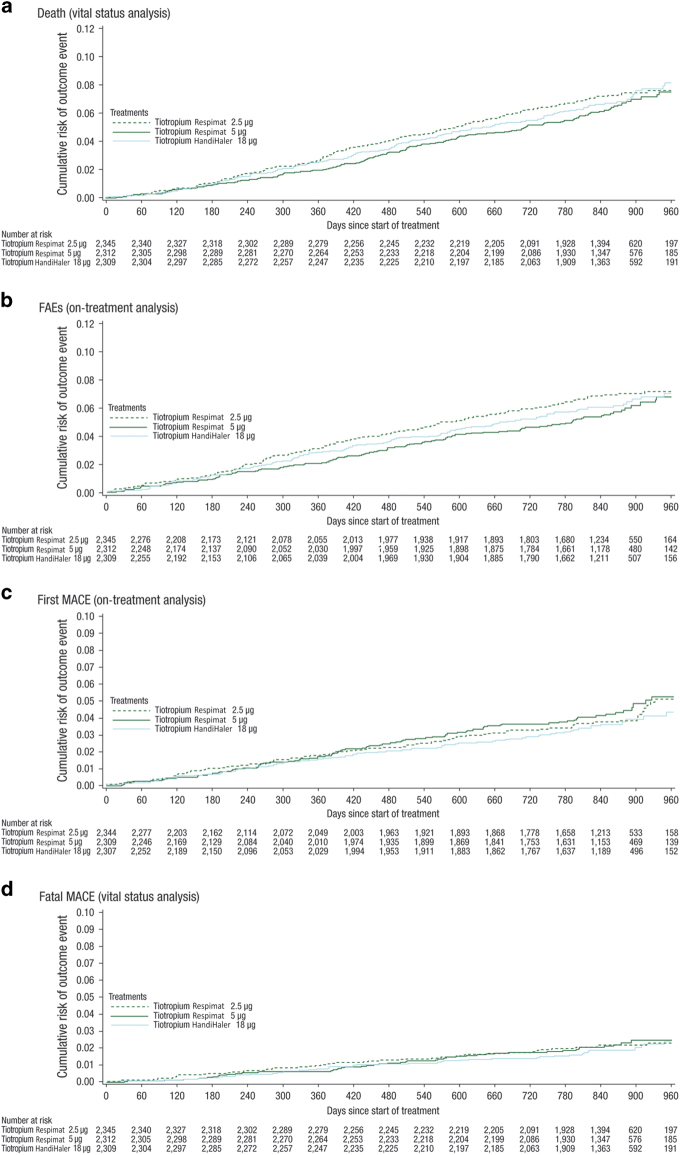
Kaplan–Meier plots showing time to (**a**) death, (**b**) FAE, (**c**) first MACE and (**d**) fatal MACE. Data for patients with event are *n* (%). CI, confidence interval; COPD, chronic obstructive pulmonary disease; FAE, fatal adverse event; MACE, major adverse cardiovascular event. Six patients (Respimat 2.5 μg, one patient; Respimat 5 μg, three patients; HandiHaler 18 μg, two patients) from sites with data irregularities were excluded from first MACE analyses. On-treatment analyses (from randomisation to drug stop+30 days).

Risks of death (measured as time to death, vital status analysis) and the adjudicated causes of death were similar between both doses of Respimat and HandiHaler 18 μg (Figures 1a, b; Table 2). Similar results were obtained for an on-treatment sensitivity analysis of time to fatal adverse event (Respimat 2.5 μg versus Respimat 5.0 μg: HR (95% CI) 1.21 (0.95, 1.54); Respimat 2.5 μg versus HandiHaler 18 μg: HR (95% CI) 1.11 (0.87, 1.40); Respimat 5 μg versus HandiHaler 18 μg: 0.91 (0.71, 1.17)). Subgroup analyses (including cardiac history at baseline and pulmonary comedication at baseline) showed no difference between groups.

Risk of first MACE (on-treatment analysis) was similar for Respimat 2.5 μg and 5.0 μg versus HandiHaler 18 μg (HR (95% CI): 1.11 (0.81, 1.51) *P*=0.523, and HR (95% CI) 1.20 (0.88, 1.63) *P*=0.244, respectively; Figure 1c). Rates of fatal MACE (vital status analysis) were similar in each of the treatment groups (Figure 1d; Table 2).

### Efficacy

**Figure 2:**
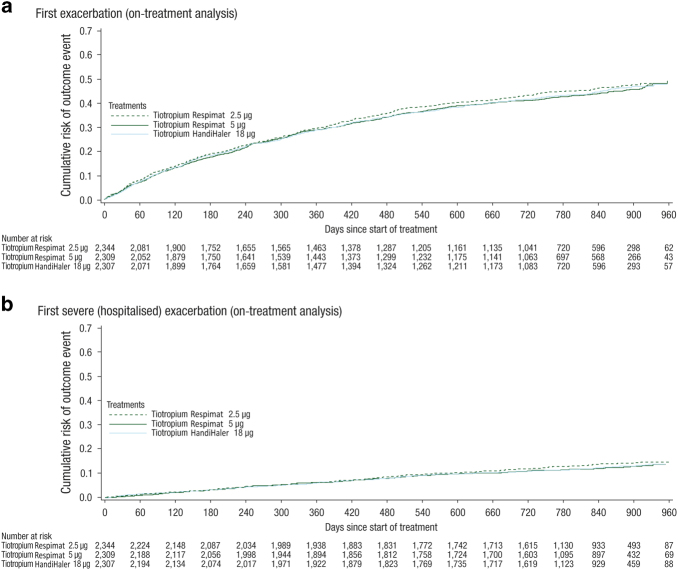
Kaplan–Meier plots showing time to (**a**) first exacerbation, and (**b**) first severe (hospitalised) exacerbation. Data for patients with event are *n* (%). COPD exacerbations were defined as the worsening of two or more major respiratory symptoms (dyspnoea, cough, sputum, chest tightness or wheezing) with a duration of at least 3 days requiring specified treatment changes: (a) mild exacerbations: a newly prescribed maintenance bronchodilator; (b) moderate exacerbations: a prescription for antibiotics, systemic glucocorticoids or both; (c) severe exacerbations: hospitalisation. Abbreviations: CI, confidence interval; COPD, chronic obstructive pulmonary disease. Six patients (Respimat 2.5 μg, one patient; Respimat 5 μg, three patients; HandiHaler 18 μg, two patients) from sites with data irregularities were excluded from analyses. On-treatment analyses (from randomisation to drug stop+1 day).

Risk of exacerbation (measured as time to first exacerbation, on-treatment analysis) showed no significant difference between the Respimat groups and HandiHaler 18 μg (Figure 2a; Table 3). The risk of moderate-to-severe and severe (hospitalised) exacerbations was also similar across groups (Figure 2b; Table 3).

Exacerbation risk did not differ between treatments in any of the patient subgroups. Patients with less impaired lung function (GOLD stage II) responded equally to Respimat and HandiHaler with respect to the rate of any exacerbation, moderate-to-severe exacerbations and severe exacerbations (Table 3). As might be expected, patients in the GOLD stage II category experienced fewer exacerbations than the overall population of anticholinergic-naive patients (Table 3).

## Discussion

### Main findings

The present study showed that patients with COPD who were naive to anticholinergic treatment at baseline, and who were treated with Respimat 2.5 or 5 μg, were at a similar risk of mortality and adverse cardiac events as patients receiving HandiHaler 18 μg.

Compared with the overall population, the present patient subgroup was similar to the TIOSPIR primary study population with regards to baseline characteristics; however, mortality rates during the study were slightly lower. It is likely that patients who were anticholinergic naive at baseline had less severe disease than the overall population; indeed, a slightly higher percentage of patients was classified as GOLD stage II. In addition, patients in the anticholinergic-naive subgroup were receiving fewer cardiovascular medications at baseline, indicating fewer cardiovascular comorbidities. Together, this may have contributed to the lower mortality rates observed. Nevertheless, the results of this *post hoc* analysis of TIOSPIR were consistent with the main study findings. In addition, the analysis addresses concerns that the safety of tiotropium Respimat could not be completely demonstrated in TIOSPIR because of a potentially selected population with a higher tolerance to anticholinergic effects at baseline.

### Interpretation of findings in relation to previously published work

COPD is a heterogeneous disease comprising a wide range of clinical phenotypes that may respond differently to treatment.^16^ Not all of these phenotypes (particularly patients with mild COPD) are represented within populations included in RCTs. Indeed, a common criticism of evidence-based medicine is that it is not known how well RCT evidence applies to COPD patients typically seen within clinical practice.^12–14^

In a comparison of primary care data from the Uncovering and Noting Long-term Outcomes in COPD (UNLOCK) database with six large RCTs of patients with COPD, significant differences were observed with respect to baseline characteristics.^13^ Patients included in the RCTs tended to be younger, were predominantly male and had significantly worse lung function and quality-of-life scores than primary care patients. Disparities were also observed with respect to exacerbations: a lower mean exacerbation rate per year and lower number of patients with ⩾1 or ⩾2 exacerbations being observed in the UNLOCK population.^13^ A further interesting observation from the UNLOCK analysis is that patients with GOLD stage I or II COPD comprised 20.7% and 53.3%, respectively, of the total UNLOCK population.^13^ This compares with a complete absence of patients at GOLD stage I and 45% of patients at GOLD stage II in the combined RCTs, normally caused by exclusion criteria for mild patients. In the TIOSPIR anticholinergic-naive subpopulation, 0.3% patients were at GOLD stage I (because of the inclusion of patients with an FEV_1_ percent predicted of <70%) and 48.8% patients were at GOLD stage II. Thus, almost half of the patients in this subgroup were considered to have moderate COPD. Comparison of other baseline characteristics from the TIOSPIR anticholinergic-naive subpopulation with those from the UNLOCK cohort and combined RCTs indicate that this subgroup may have greater similarity to the UNLOCK population than other large COPD trials. Fewer patients in the anticholinergic subgroup had also experienced one or more exacerbation in the year before the study (48%) than patients from other large RCTs (59%), thus better reflecting the UNLOCK population (44%; Supplementary Table 1). Together, these comparisons indicate that the anticholinergic-naive subgroup from TIOSPIR may better reflect patients observed in primary care than patients typically randomised to other large COPD trials.

The primary efficacy outcome from TIOSPIR was time to first COPD exacerbation. Exacerbations are an important component of COPD, having a significant impact on disease progression and health status, as well as increasing the risk of further exacerbations and death.^17–19^ Thus, the prevention of exacerbations is a primary goal in COPD management.^20^ Tiotropium Respimat and HandiHaler have been shown to reduce COPD exacerbations versus placebo and other therapies, including among subgroups of patients.^6,21–28^ For example, in a prespecified secondary analysis of the Understanding Potential Long-term Impacts on Function with Tiotropium (UPLIFT) trial, tiotropium HandiHaler reduced the number of exacerbations per patient-year by 16% versus control in patients naive to maintenance therapy before the study.^25^ In subgroup analyses of the Prevention Of Exacerbations with Tiotropium in COPD (POET-COPD) study, tiotropium HandiHaler significantly prolonged the time to first exacerbation (HR (95% CI), 0.88 (0.79, 0.99); *P*=0.028) and first severe exacerbation (HR (95% CI), 0.66 (0.48, 0.91); *P*=0.012) in GOLD stage II patients, and significantly reduced the annual exacerbation rate (rate ratio [95% CI], 0.77 (0.63, 0.94); *P*=0.012) in maintenance therapy–naive patients, when compared with salmeterol.^27^ Before TIOSPIR, the relative efficacy of exacerbation reduction by Respimat and HandiHaler was unknown. In this current *post hoc* analysis, the incidence of exacerbations was shown to be similar among anticholinergic-naive patients who were treated with either Respimat or HandiHaler. Furthermore, the subgroup analysis of exacerbation risk in GOLD stage II patients shows that Respimat and HandiHaler have similar efficacy profiles in patients with less impaired lung function or health status.

Further evidence for tiotropium’s safety was provided by the low incidence of discontinuations observed in each of the treatment groups because of anticholinergic side effects. Other clinical studies of tiotropium in COPD have reported similarly low rates of discontinuation because of undesirable effects such as dry mouth, occurring in 0.1% of patients treated with tiotropium Respimat 5 μg (5 clinical trials)^2^ and 0.2% of patients treated with tiotropium HandiHaler 18 μg (28 clinical trials).^1^

### Strengths and limitations of this study

A key limitation of the study is that it was a *post hoc* analysis rather than a prespecified subgroup analysis. Nevertheless, being one of the largest, long-term, randomised trials of a broad range of patients with COPD, TIOSPIR lends itself to statistically powerful subgroup analyses.

### Implications for future research, policy and practice

This *post hoc* analysis examined whether the subpopulation of patients in TIOSPIR who were naive to anticholinergic therapy at baseline responded differently to treatment with tiotropium Respimat than to tiotropium HandiHaler. Patients who are naive to anticholinergic therapy are frequently seen within daily practice, and the results of this analysis will therefore be of particular interest to primary care physicians and policy makers. Better definition of treatment responses of individuals according to their treatment history will ultimately help tailor more targeted interventions in patients with COPD.

### Conclusions

In this *post hoc* analysis, patients with COPD who were naive to anticholinergic treatment had similar efficacy and safety profiles when treated with tiotropium Respimat or HandiHaler in the TIOSPIR trial.

This study provides further data that addresses prior safety concerns regarding tiotropium Respimat and provides additional evidence that tiotropium Respimat treatment is also safe for patients who were not previously treated with an anticholinergic, including patients with mild disease more commonly seen in primary care.

## Figures and Tables

**Table 1:** Patient disposition and baseline characteristics in anticholinergic-naive patients

*Variable*	*Tiotropium Respimat* 2.5 *μg (*n*=2,344)*	*Tiotropium Respimat* *5 μg (*n*=2,309)*	*Tiotropium HandiHaler* *18 μg (*n*=2,307)*
*Gender, n (%)*			
Male	1,686 (71.9)	1,694 (73.4)	1,641 (71.1)
Mean age, years (s.d.)	64.1 (9.3)	64.0 (9.2)	64.1 (9.2)
BMI, mean kg/m^2^ (s.d.)	26.1 (5.7)	26.0 (5.7)	26.1 (5.8)
Smoking history, mean pack-years (s.d.)	42.2 (24.3)	42.5 (23.9)	41.6 (23.6)
			
*Post-bronchodilator spirometry, mean (s.d.)*			
FEV_1_, L	1.365 (0.495)	1.374 (0.494)	1.360 (0.488)
FEV_1_, % predicted	48.7 (14.1)	48.8 (13.9)	48.8 (14.2)
FVC, L	2.688 (0.835)	2.711 (0.840)	2.685 (0.846)
Ratio of FEV_1_ to FVC	0.512 (0.116)	0.511 (0.114)	0.511 (0.113)
			
*GOLD stage, n (%)*			
I	4 (0.2)	10 (0.4)	7 (0.3)
II	1,142 (48.7)	1,112 (48.2)	1,140 (49.4)
III	896 (38.2)	908 (39.3)	868 (37.6)
IV	261 (11.1)	238 (10.3)	263 (11.4)
			
*mMRC score, n (%)*			
0 (including not breathless)	186 (8.0)	200 (8.7)	216 (9.4)
1	923 (39.5)	871 (37.8)	900 (39.1)
2	841 (36.0)	832 (36.1)	813 (35.3)
3	350 (15.0)	370 (16.1)	331 (14.4)
4	38 (1.6)	32 (1.4)	42 (1.8)
			
*Patients with exacerbations in the previous year, n (%)*			
0	1,210 (51.6)	1,203 (52.1)	1,191 (51.6)
1	654 (27.9)	653 (28.3)	666 (28.9)
⩾2	477 (20.3)	451 (19.5)	448 (19.4)
			
Previous cardiac arrhythmia, n (%)	208 (8.9)	212 (9.2)	194 (8.4)
Previous MI, n (%)	118 (5.0)	122 (5.3)	117 (5.1)
Previous stroke, n (%)	53 (2.3)	56 (2.4)	46 (2.0)
Previous IHD or CAD, n (%)	346 (14.8)	334 (14.5)	333 (14.4)
Taking CV medication, n (%)	1,109 (47.3)	1,062 (46.0)	1,061 (46.0)
Use of respiratory medication, n (%)	1,830 (78.1)	1,764 (76.4)	1,785 (77.4)
LABA	1,195 (51.0)	1,177 (51.0)	1,219 (52.8)
SABA	1,076 (45.9)	1,061 (46.0)	1,052 (45.6)
ICS	1,167 (49.8)	1,152 (49.9)	1,175 (50.9)

Abbreviations: BMI, body mass index; CAD, coronary artery disease; CV, cardiovascular; FEV_1_, forced expiratory volume in 1 s; FVC, forced vital capacity; GOLD, Global Initiative for Chronic Obstructive Lung Disease; ICS, inhaled corticosteroid; IHD, ischaemic heart disease; LABA, long-acting β_2_-agonist; MI, myocardial infarction; mMRC, modified Medical Research Council; SABA, short-acting β_2_-agonist; s.d, standard deviation.

Six patients (Respimat 2.5 μg, one patient; Respimat 5 μg, three patients; HandiHaler 18 μg, two patients) from sites with data irregularities were excluded.

**Table 2:** Incidence of adjudicated causes of mortality and fatal MACE in anticholinergic-naive patients

*Variable*	*Tiotropium Respimat* *2.5 μg (*n*=2,345)*	*Tiotropium Respimat* *5 μg (*n*=2,312)*	*Tiotropium HandiHaler* *18 μg (*n*=2,309)*	*Rate ratio (95% CI)*	
				*Tiotropium Respimat* *2.5 μg vs. HandiHaler*	*Tiotropium Respimat* *5 μg vs. HandiHaler*
Total patients with deaths	169 (3.2)	149 (2.8)	159 (3.0)	1.05 (0.84, 1.30)	0.93 (0.75, 1.17)
Patients with fatal MACE	49 (0.9)	49 (0.9)	43 (0.8)	1.12 (0.75, 1.69)	1.14 (0.75, 1.71)
*Adjudicated causes of death*					
Cardiac disorders	10 (0.2)	13 (0.2)	5 (0.1)	1.97 (0.67, 5.77)	2.59 (0.92, 7.27)
General disorders including sudden (cardiac) death	53 (1.0)	41 (0.8)	46 (0.9)	1.14 (0.77, 1.69)	0.89 (0.58, 1.35)
Neoplasms benign, malignant and unspecified	44 (0.8)	29 (0.5)	32 (0.6)	1.36 (0.86, 2.14)	0.90 (0.55, 1.49)
Respiratory, thoracic and mediastinal disorders	36 (0.7)	34 (0.6)	36 (0.7)	0.99 (0.62, 1.57)	0.94 (0.59, 1.50)
COPD (PT)	34 (0.6)	31 (0.6)	34 (0.6)	0.99 (0.61, 1.59)	0.91 (0.56, 1.48)
Infections and infestations	10 (0.2)	10 (0.2)	13 (0.2)	0.76 (0.33, 1.73)	0.77 (0.34, 1.75)
Gastrointestinal disorders	3 (0.1)	9 (0.2)	8 (0.2)	0.37 (0.10, 1.39)	1.12 (0.43, 2.91)

Abbreviations: CI, confidence interval; COPD, chronic obstructive pulmonary disease; MACE, major adverse cardiovascular event; MedDRA, Medical Dictionary for Regulatory Activities; PT, preferred term; SOC, system organ class. Data shown are *n* (rate per 100 patient-years). Risk-adjusted rates of adjudicated causes of death by treatment, MedDRA (version 16.0, MedDRA MSSO, McLean, VA, USA) SOC and PT. Vital status analysis. MACE included stroke, myocardial infarction, sudden death, cardiac death, sudden cardiac death or fatal event in the SOCs for cardiac and vascular disorders.

**Table 3:** Risk of exacerbation in anticholinergic-naive patients

*Variable*	*Tiotropium Respimat* *2.5 μg (*n*=2,344)*	*Tiotropium Respimat* *5 μg (*n*=2,309)*	*Tiotropium HandiHaler* *18 μg (*n*=2,307)*	*Hazard ratio (95% CI);* P*-value*	
				*Tiotropium Respimat* *2.5 μg versus HandiHaler*	*Tiotropium Respimat* *5 μg versus HandiHaler*
*All patients*					
Any exacerbation					
Patients with event, *n* (%)	979 (41.8)	923 (40.0)	948 (41.1)	1.04 (0.95, 1.14); *P*=0.359	0.99 (0.90, 1.08); *P*=0.829
No. of events	2,022	2,002	1,955		
Adjusted rate of events/patient-year (95% CI)	0.45 (0.42, 0.48)	0.45 (0.42, 0.48)	0.44 (0.41, 0.47)		
Moderate-to-severe exacerbation					
Patients with event, *n* (%)	957 (40.8)	902 (39.1)	926 (40.1)	1.04 (0.95, 1.14); *P*=0.364	0.99 (0.90, 1.09); *P*=0.840
No. of events	1,968	1,956	1,902		
Adjusted rate of events/patient-year (95% CI)	0.43 (0.40, 0.47)	0.44 (0.41, 0.47)	0.43 (0.40, 0.46)		
Severe (hospitalised) exacerbations					
Patients with event, *n* (%)	282 (12.0)	250 (10.8)	255 (11.1)	1.10 (0.93, 1.30); *P*=0.277	0.99 (0.83, 1.18); *P*=0.917
No. of events	406	376	363		
Adjusted rate of events/patient-year (95% CI)	0.09 (0.08, 0.11)	0.09 (0.08, 0.10)	0.08 (0.07, 0.10)		
*GOLD stage II subgroup*					
Any exacerbation					
Patients with event, *n* (%)	442 (38.6)	391 (34.8)	426 (37.1)	1.05 (0.92, 1.20); *P*=0.472	0.95 (0.82, 1.08); *P*=0.422
No. of events	851	809	837		
Adjusted rate of events/patient-year (95% CI)	0.38 (0.34, 0.42)	0.37 (0.34, 0.42)	0.38 (0.34, 0.42)		
Moderate-to-severe exacerbation					
Patients with event, *n* (%)	428 (37.3)	380 (33.9)	415 (36.2)	1.04 (0.91, 1.19); *P*=0.546	0.94 (0.82, 1.08); *P*=0.410
No. of events	822	781	817		
Adjusted rate of events/patient-year (95% CI)	0.36 (0.33, 0.40)	0.36 (0.32, 0.40)	0.37 (0.33, 0.41)		
Severe (hospitalised) exacerbations					
Patients with event, *n* (%)	84 (7.3)	61 (5.4)	78 (6.8)	1.07 (0.79, 1.46); *P*=0.650	0.81 (0.58, 1.13); *P*=0.206
No. of events	116	84	113		
Adjusted rate of events/patient-year (95% CI)	0.05 (0.04, 0.07)	0.04 (0.03, 0.05)	0.05 (0.04, 0.07)		

Abbreviations: CI, confidence interval; GOLD, Global Initiative for Chronic Obstructive Lung Disease.

On-treatment analysis (from randomisation to drug stop+1 day). Six patients (Respimat 2.5 μg, one patient; Respimat 5 μg, three patients; HandiHaler 18 μg, two patients) from sites with data irregularities were excluded.
