# Supplementary material for: Safety and efficacy of tiotropium Respimat versus HandiHaler in patients naive to treatment with inhaled anticholinergics: a post hoc analysis of the TIOSPIR trial
Source: NPJ Prim Care Respir Med. 2015 Nov 5;25:15067–. doi: 10.1038/npjpcrm.2015.67 (PMC4634206; doi:10.1038/npjpcrm.2015.67)
Supplement: Supplementary Table 1 [file npjpcrm201567-s1.doc]

**Supplementary Table 1.** Baseline comparison of the UNLOCK studies versus anticholinergic naïve patients from TIOSPIR®

| ***Characteristic*** | ***UNLOCK studies (N = 3,508)*** | ***Large COPD studies^a^ (N = 23,860)*** | ***TIOSPIR anticholinergic naïve  (N = 6,960)*** |
| --- | --- | --- | --- |
| Age, years | 66.1 | 63.7 | 64.1 |
| Male, % | 60.9 | 73.3 | 72.1 |
| Current smokers, %  Pack years | 42.9  43.6 | 40.7  44.9 | 43.0  42.1 |
| BMI, kg/m^2^ | 26.3 | 25.6 | 26.1 |
| Postbronchodilator FEV_1_, % predicted | 63.8 | 47.4 | 48.8 |
| FEV_1_:FVC, % | 55.7 | 46.5 | 51.1 |
| GOLD distribution, %  Mild (GOLD I)  Moderate (GOLD II)  Severe (GOLD III)  Very severe (GOLD IV) | 20.7  53.3  21  5.8 | –  45.0  44.5  11.5 | 0.3^b^  48.8  38.4  10.9 |
| Exacerbation history, %  Patients with ≥ 1 exacerbation in preceding year | 44 | 59 | 48 |
| Patients with ≥ 2 exacerbation in preceding year | 22 | 30 | 19 |

Data are overall mean values, in which every dataset or study contributed equally to the overall means.

^a^Studies included ECLIPSE, ISOLDE, POET-COPD®, TORCH, TRISTAN, UPLIFT®. ^b^Patients with GOLD stage I were excluded from tiotropium studies. Table adapted from *Kruis et al*.^13^

Abbreviations: BMI, body mass index; ECLIPSE, Evaluation of COPD Longitudinally to Identify Predictive Surrogate Endpoints; FEV_1_, forced expiratory volume in 1 second; FVC, forced vital capacity; GOLD, Global Initiative for Chronic Obstructive Lung Disease; ISOLDE, Inhaled Steroids in Obstructive Lung Disease in Europe; POET-COPD®, Prevention of Exacerbations with Tiotropium-COPD; TORCH, Towards a Revolution in COPD Health; TRISTAN, Trial of Inhaled Steroids and Long-acting β2 Agonists; UNLOCK, Uncovering and Noting Long-term Outcomes in COPD; UPLIFT®, Understanding Potential Long-term Impacts on Function with Tiotropium.
